# Fractional-order numerical modeling to study chloride ion transport in concrete with fly ash or slag additions

**DOI:** 10.1371/journal.pone.0294858

**Published:** 2023-11-30

**Authors:** Li Zhou, Guangdong Huang, Ruige Chen

**Affiliations:** School of Science, China University of Geosciences, Beijing, China; Visvesvaraya National Institute of Technology, INDIA

## Abstract

To better study the chloride ion migration in concrete with fly ash or ground granulated blast furnace slag under low fatigue load, a Caputo time fractional-order chloride diffusion model is developed in this paper. The model, grounded in Fick’s second law with a fractional-order derivative, employs an implicit numerical method for discretization, resulting in a fractional-order numerical scheme. The stability and convergence of the scheme are rigorously proven within the paper. The model’s unknown parameters are estimated using genetic algorithm with a grid method. To validate the model’s effectiveness, its numerical solution is juxtaposed with experimental results from chloride erosion studies. Furthermore, the fitting efficacy of the Caputo time fractional-order numerical scheme is compared with that of the classical Fick’s second law numerical scheme and analytical solution. The research findings demonstrate that the fractional-order numerical scheme can more accurately simulate the chloride concentration in concrete containing fly ash or slag. Additionally, the model shows promise in predicting the service life of fly ash or slag concrete.

## Introduction

Many reinforced concrete structures in coastal areas are subjected to wetting-drying cycles or total submersion. The reinforcement in these concretes subjected to chloride salt attack reduces the concrete structures’ service life [1]. Moreover, for concrete structures such as port terminals and bridges, they will also bear the repeated action of traffic loads. Under the action of alternating loads, these concrete structures are subjected to specific fatigue damage. The internal structure of the concrete subjected to fatigue loading undergo cracks and microstructural changes, which can increase the rate of chloride ion penetration and accelerate the corrosion of reinforcement in concrete [2]. Therefore, it is economically and practically essential to study the improvement of durability of fatigue-damaged concrete and to reduce the diffusion of external chloride ions into fatigue-damaged concrete [3].

There are already many practical and environmentally friendly methods to improve the durability of reinforced concrete. These methods include: changing the formulation of concrete to manufacture new types of concrete [4, 5], coating concrete reinforcement with protective layers [6, 7], and adding polymer modifiers [8–11], rubber [12], and minerals [13, 14] to concrete. The use of Class F fly ash or finely ground blast furnace slag as admixtures in concrete is convenient, relatively low-cost, and environmentally friendly [15]. Moreover, these minerals can significantly reduce the porosity of the concrete, thereby reducing the permeability of the concrete and enhancing its durability [16]. Ahmet et al. [17] experimentally tested the permeability of chloride ions in concrete with different proportions of fly ash. The results showed that the concrete with 15% fly ash has better corrosion resistance. Ning et al. [18] experimentally explored how adding mineral admixture improves the chloride ion permeability resistance of concrete. Although the numerical values obtained through experimental methods are relatively accurate, they are discrete. In contrast, numerical simulation methods yield continuous data, effectively simulating the distribution of chloride ions in concrete structures. Liu et al. [19] have developed a numerical model that takes into account the porosity of concrete, chloride ion binding, and electrochemical coupling among multiple species, enabling the prediction of the service life of alkali-activated fly ash/slag concrete. In the conventional model, chloride ion diffusion is typically assumed to follow Fick’s second law, under the premise that the cement matrix is inert and uniformly porous. However, numerous experimental and field observations indicate that chloride ions exhibit anomalous diffusion in concrete [20]. This anomalous diffusion, which demonstrates time-dependency, violates Fick’s second law. Consequently, conventional models fail to account for the time-dependent nature of chloride ion anomalous diffusion. The power-law model with time-varying coefficients can address this shortcoming. Zhang et al. [21] have developed a power-law model with time-varying coefficients to capture the time-dependent permeability of chloride ions in fly ash concrete.

These models are constructed upon the time-integer order derivative diffusion equation derived from Fick’s second law. While integer order differential equations consider instantaneous changes and have their merits, they also possess inherent limitations. The advent of fractional order differential equations has broadened the scope of applications, particularly in the realm of material transport within non-uniform mixed media. The distinctive temporal and spatial memory effects inherent to fractional order differential equations have yielded superior outcomes in characterizing material transport issues within non-uniform mixed media, an achievement beyond the reach of integer order differential equations. Therefore, employing fractional order differential equations to study chloride ion transport not only accounts for instantaneous changes but also provides a holistic view of the entire process. This approach offers a more authentic representation of the chloride ion transport process within concrete, effectively addressing the localized limitations of integer order differential equations. Despite these advancements, there remains a gap in the literature. To date, no studies have explored the migration characteristics of chloride ions in mineral-added concrete using a time-fractional numerical model.

Therefore, the novelty of this paper is to establish the Caputo time fractional-order diffusion equation based on the classical Fick’s second law. Using an implicit numerical method discretizes the equation to establish a fractional-order numerical scheme to find the numerical solution. Then we further analyze chloride ions’ migration and corrosion inhibition mechanism in concrete with fly ash addition and slag addition in two conditions (wetting-drying cycles and complete submersion) by comparing the distribution plots. Furthermore, we verify that the Caputo time fractional-order numerical scheme is more consistent with the numerical results of the actual data than the numerical scheme and analytical solution fit of the integer-order Fick’s second law.

## Theoretical model of fractional-order transport of chloride ions

### Mathematical models

We established the Caputo time fractional-order diffusion equation by introducing the Caputo time fractional derivatives based on Fick’s second law. The equation [22, 23]:

$$\begin{array}{cc} {}_{0}^{C}\;D_{t}^{\alpha }u\left(x,t\right)=\;D\frac{\partial^{2}u\left(x,t\right)}{\partial x^{2}}. & \;0\leq x\leq b,\;0<t\leq T, \end{array}$$ (1)

under the initial condition:

$$\begin{array}{c} u(x,0)=0,\;\;0\leq x\leq b, \end{array}$$ (2)

and the boundary conditions:

$$\begin{array}{c} u\left(0,t\right)=\varphi_{0}\left(t\right),\;\;u\left(b,t\right)=0,\;0<t\leq T. \end{array}$$ (3)

Where *u* is the free chloride ion content, *D* is the chloride ion diffusion coefficient, and *t* is the immersion time. Moreover, *x* is the diffusion distance of chloride ions (*x*-direction), the Caputo time fractional derivative of order 0 < *α* < 1.

### Numerical model

Using the finite difference method, we establish a fully discrete numerical format for model (1). We define *t*_*k*_ = *kτ*, *k* = 0, 1, …, *n*; *x*_*i*_ = *ih*, *i* = 0, 1, …, *m*, where *τ* = *T*/*n* and *h* = *b*/*m* are time and space step sizes, respectively. Assume that $u\left(x,t\right)\in C_{x,t}^{4,2}\left(\left[0,b\right]\times \left[0,T\right]\right).$ We discretize the Caputo time-fractional order derivatives using the *L*1-algorithm [24, 25]:

$$\begin{array}{c} {}_{0}^{C}\;D_{t}^{\alpha }u\left(x,t_{k+1}\right)=\frac{\tau^{-\alpha }}{\Gamma (2-\alpha )}\sum_{j=0}^{k}b_{j}^{\left(\alpha \right)}\left[u\left(x,t_{k+1-j}\right)-u\left(x,t_{k-j}\right)\right]+\;O\left(\tau^{2-\alpha }\right), \end{array}$$ (4)

where $b_{j}^{\left(\alpha \right)}={\left(j+1\right)}^{1-\alpha }-j^{1-\alpha },\;j=0,1,\ldots ,n,\;i=1,2,\ldots ,p.$

Then, we use the central difference formula to discretize second-order space derivative:

$$\begin{array}{c} \frac{\partial^{2}}{\partial x^{2}}u\left(x_{i},t\right)=\frac{u\left(x_{i+1},t\right)-2u\left(x_{i},t\right)+u\left(x_{i-1},t\right)}{h^{2}}+\;O\left(h^{2}\right). \end{array}$$ (5)

Hence we have

$$\begin{array}{c} \begin{array}{cc} & \frac{1}{\Gamma \left(2-\alpha \right)\tau^{\alpha }}\sum_{j=0}^{k}b_{j}^{\left(\alpha \right)}\left[u\left(x_{i},t_{k-j+1}\right)-u\left(x_{i},t_{k-j}\right)\right]\\ = & \;D\frac{u\left(x_{i+1},t_{k+1}\right)-2u\left(x_{i},t_{k+1}\right)+u\left(x_{i-1},t_{k+1}\right)}{h^{2}}+R_{i,k+1}, \end{array} \end{array}$$ (6)

where

$$|\;R_{i,k+1}|=C\left(\tau^{2-\alpha }+h^{2}\right),$$

*C* is a constant.

Let $u_{i}^{k}$ be the numerical approximation to *u*(*x*_*i*_, *t*_*k*_). Following are the implicit difference approximations of Eq (1):

$$\begin{array}{c} \begin{array}{c} \frac{1}{\Gamma \left(2-\alpha \right)\tau^{\alpha }}\sum_{j=0}^{k}b_{j}^{\left(\alpha \right)}\left[u_{i}^{k-j+1}-u_{i}^{k-j}\right]=\;D\frac{u_{i+1}^{k+1}-2u_{i}^{k+1}+u_{i-1}^{k+1}}{h^{2}}. \end{array} \end{array}$$ (7)

Set $\mu =\frac{\Gamma (2-\alpha )}{\tau^{1-\alpha }},r=\;D\tau h^{-2}.$ The differential form of Eqs (1)-(3) is as follows:

$$\begin{array}{c} \begin{array}{c} \left(\mu^{-1}+2r\right)u_{i}^{k+1}-r\left(u_{i+1}^{k+1}-u_{i-1}^{k+1}\right)=\mu^{-1}\sum_{j=0}^{k-1}\left(b_{j}^{\left(\alpha \right)}-b_{j+1}^{\left(\alpha \right)}\right)u_{i}^{k-j}+\mu^{-1}b_{k}^{\alpha }u_{i}^{0},\\ i=1,2,\ldots ,m-1;k=1,2,\ldots ,n-1. \end{array} \end{array}$$ (8)

$$\begin{array}{c} \begin{array}{c} u_{i}^{0}=0,i=0,1,\ldots ,m,\\ u_{0}^{k}=\varphi_{0}\left(t_{k}\right),u_{m}^{k}=0,k=1,2,\ldots ,n. \end{array} \end{array}$$ (9)

We convert (8) to the following format to simplify the study:

$$\begin{array}{c} \begin{array}{c} \left(1+2r\mu \right)u_{i}^{k+1}-r\mu \left(u_{i+1}^{k+1}-u_{i-1}^{k+1}\right)=u_{i}^{k}-\sum_{j=1}^{k}b_{j}^{\left(\alpha \right)}\left(u_{i}^{k-j+1}-u_{i}^{k-j}\right),\\ i=1,2,\ldots ,m-1;k=1,2,\ldots ,n-1. \end{array} \end{array}$$ (10)

Then here is another way to write the numerical model:

$$\begin{array}{c} \begin{array}{c} \;AU^{k+1}=U^{k}-\sum_{j=1}^{k}b_{j}^{\left(\alpha \right)}\left(U^{k-j+1}-U^{k-j}\right)+r\mu u_{0}^{k+1}\;E,\\ i=1,2,\ldots ,m-1;k=1,2,\ldots ,n-1, \end{array} \end{array}$$ (11)

where *A* = [*I* − *rμC*],

$$\begin{array}{c} \;C=(\begin{array}{ccccc} -2 & 1 & & & \\ 1 & -2 & 1 & \\ & \ddots & \ddots & \ddots & \\ & & 1 & -2 & 1\\ & & & 1 & -2 \end{array}), \end{array}$$

$\;E=\left(1,0,\ldots ,0\right),U^{k}={\left(u_{1}^{k},u_{2}^{k},\ldots ,u_{m-1}^{k}\right)}^{T}.$

## Stability of fractional-order numerical models

Here we discuss the fractional-order numerical model (10), which has initial and boundary conditions $u_{i}^{0}=0,i=0,1,\ldots ,m,u_{0}^{k}=\varphi_{0}\left(t_{k}\right),u_{m}^{k}=0,k=1,2,\ldots ,n.$

**Lemma 1** The coefficients $b_{j}^{\left(\alpha \right)},j=0,1,\ldots ,$ satisfy:

1.
$b_{0}^{\left(\alpha \right)}=1,b_{j}^{\left(\alpha \right)}>0,j=1,2,\ldots ;$

2.
$b_{j}^{\left(\alpha \right)}>b_{j+1}^{\left(\alpha \right)},j=1,2,\ldots .$

We’ll discuss the stability of numerical method (10). Let $\left|\right|u^{k+1}{\left|\right|}_{\infty }={\text{max}}_{1\leq i\leq m-1}\left|u_{i}^{k+1}\right|$

**Theorem 1** *The fractional-order numerical method* (10) *is unconditionally stable*.

*Proof.* Let ${\tilde{u}}_{i}^{k}$ (0 ≤ *i* ≤ *m*;0 ≤ *j* ≤ *n*) be the approximate solution of (10), the error $\varepsilon_{i}^{k}={\tilde{u}}_{i}^{k}-u_{i}^{k}$ (0 ≤ *i* ≤ *m*;0 ≤ *j* ≤ *n*) satisfies:

$$\begin{array}{c} \begin{array}{c} \left(1+2r\mu \right)\varepsilon_{i}^{k+1}-r\mu \left(\varepsilon_{i+1}^{k+1}-\varepsilon_{i-1}^{k+1}\right)=\varepsilon_{i}^{k}-\sum_{j=1}^{k}b_{j}^{\left(\alpha \right)}\left(\varepsilon_{i}^{k-j+1}-\varepsilon_{i}^{k-j}\right)\\ i=1,2,\ldots ,m-1;k=1,2,\ldots ,n-1. \end{array} \end{array}$$ (12)

By Lemma 1, we have

$$\begin{array}{c} \;\left(\mu^{-1}\right)\left|\right|u^{k+1}{\left|\right|}_{\infty }=\left|\mu^{-1}u_{i_{0}}^{k+1}\right|\\ =\left(\mu^{-1}+2r-r-r\right)\left|u_{i_{0}}^{k+1}\right|\\ \leq \left(\mu^{-1}+2r\right)|u_{i_{0}}^{k+1}\left|-r\right|u_{i_{0}+1}^{k+1}\left|-r\right|u_{i_{0}-1}^{k+1}|\\ \leq |\left(\mu^{-1}+2r\right)u_{i_{0}}^{k+1}-ru_{i_{0}+1}^{k+1}-ru_{i_{0}-1}^{k+1}|\\ =|\mu^{-1}\sum_{j=1}^{k}\left(b_{k-j}^{\left(\alpha \right)}-b_{k-j+1}^{\left(\alpha \right)}\right)u_{i_{0}}^{j}]. \end{array}$$

since *μ*^−1^ > 0, we have

$$\begin{array}{c} \begin{array}{c} |u_{i_{0}}^{k+1}\left|\leq \right|\sum_{j=1}^{k}\left(b_{k-j}^{\left(\alpha \right)}-b_{k-j+1}^{\left(\alpha \right)}\right)u_{i_{0}}^{j}]. \end{array} \end{array}$$ (13)

For *j* = 2, we assume that $|u_{i_{0}}^{k+1}|={\text{max}}_{1\leq i\leq m-1}\left|u_{i}^{k+1}\right|$. Applying (13), we have

$$|u_{i_{0}}^{2}\left|\leq \right|\left(b_{0}^{\left(\alpha \right)}-b_{1}^{\left(\alpha \right)}\right)u_{i_{0}}^{1}|=u_{i_{0}}^{1}.$$

Suppose that

$$\left|\right|u^{j}{\left|\right|}_{\infty }\leq u_{i_{0}}^{1},2\leq j\leq k.$$

When *j* = *k* + 1, we obtain

$$\begin{array}{c} \begin{array}{c} \;\left|\right|u^{k+1}{\left|\right|}_{\infty }\leq \left|\sum_{j=1}^{k}\left(b_{k-j}^{\left(\alpha \right)}-b_{k-j+1}^{\left(\alpha \right)}\right)u_{i_{0}}^{j}\right|\\ \leq |\sum_{j=1}^{k}\left(b_{k-j}^{\left(\alpha \right)}-b_{k-j+1}^{\left(\alpha \right)}\right)\left||\right|u^{1}{\left|\right|}_{\infty }\\ \leq |\left(b_{0}^{\left(\alpha \right)}-b_{1}^{\left(\alpha \right)}\right)\left||\right|u^{1}{\left|\right|}_{\infty }\\ =\left|\right|u^{1}{\left|\right|}_{\infty }. \end{array} \end{array}$$ (14)

So, ||*u*^*k*+1^||_∞_ ≤ ||*u*^1^||_∞_ applying (12), we have

$$\left|\right|E^{k}{\left|\right|}_{\infty }\leq \left|\right|E^{0}{\left|\right|}_{\infty },k=1,2,\ldots ,n,$$

where $\left|\right|E^{k}{\left|\right|}_{\infty }={\text{max}}_{1\leq i\leq m-1}\left|\varepsilon_{i}^{k}\right|.$ The numerical method defined by (10) is unconditionally stable.

## Convergence of fractional-order numerical models

Here we will discuss the convergence of the fractional-order numerical model (10).

**Theorem 2** *Let* $u_{i}^{k}$ *be the numerical solution of the* Eq (10), *u*(*x*_*i*_, *t*_*k*_) *is the solution of the problem(1)-(3). The fractional-order numerical model* (10) *is convergent. That is, there is a positive constant* *C*, *such that* $|u_{i}^{k}-u\left(x_{i},t_{k}\right)|\leq C\left(\tau^{2-\alpha }+h\right)$, *where* *i* = 1, 2, …,*m* − 1; *k* = 1, 2, …, *n*.

*Proof.* Let *u*(*x*_*i*_, *t*_*k*_), (0 ≤ *i* ≤ *m*;0 ≤ *j* ≤ *n*) be the exact solution of the Eqs (1)-(3) at mesh point (*x*_*i*_, *t*_*k*_). Define $\eta_{i}^{k}=u\left(x_{i},t_{k}\right)-u_{i}^{k},\left(0\leq i\leq m-1;1\leq j\leq n\right)$ and $Y^{k}=\left(\eta_{1}^{k},\eta_{2}^{k},\ldots ,\eta_{m-1}^{k}\right)$. Subtracting (10) from (6), we obtain

$$\begin{array}{c} \begin{array}{cc} & \left(1+2r\mu \right)\eta_{i}^{k+1}-r\mu \left(\eta_{i+1}^{k+1}-\eta_{i-1}^{k+1}\right)\\ = & \eta_{i}^{k}-\sum_{j=1}^{k}b_{j}^{\left(\alpha \right)}\left(\eta_{i}^{k-j+1}-\eta_{i}^{k-j}\right)+\mu \tau R_{i}^{k+1},\\ & \;i=1,2,\ldots ,m-1;k=1,2,\ldots ,n-1. \end{array} \end{array}$$ (15)

Let$|Y_{i_{0}}^{k+1}|={\text{max}}_{1\leq i\leq m-1}\left|Y_{i}^{k+1}\right|$, we have

$$\begin{array}{c} \begin{array}{c} \;\left|\right|Y^{k+1}{\left|\right|}_{\infty }=\left|Y_{i_{0}}^{k+1}\right|\\ =\left(1+2r\mu -r\mu -r\mu \right)|Y_{i_{0}}^{k+1}|\\ \leq \left(1+2r\mu \right)|Y_{i_{0}}^{k+1}\left|-r\mu \right|Y_{i_{0}+1}^{k+1}\left|-r\mu \right|Y_{i_{0}+1}^{k+1}|\\ \leq |\left(1+2r\mu \right)Y_{i_{0}}^{k+1}-r\mu Y_{i_{0}+1}^{k+1}-r\mu Y_{i_{0}+1}^{k+1}|\\ =|\sum_{j=1}^{k}\left(b_{k-j}^{\left(\alpha \right)}-b_{k-j+1}^{\left(\alpha \right)}\right)Y_{i_{0}}^{j}+\mu \tau R_{i}^{k+1}|. \end{array} \end{array}$$ (16)

For *j* = 1, applying (16), we have

$$|Y_{i_{0}}^{1}|\leq |\mu \tau R_{i}^{k+1}|={\left(b_{0}^{\left(\alpha \right)}\right)}^{-1}\mu \tau ||R^{1}{\left|\right|}_{\infty }.$$

Suppose that

$$\left|\right|Y^{j}{\left|\right|}_{\infty }\leq {\left(b_{j}^{\left(\alpha \right)}\right)}^{-1}{\mu \tau ||R||}_{\infty }.2\leq j\leq k.$$

When *j* = *k* + 1, we obtain

$$\begin{array}{c} \begin{array}{cc} & \left|\right|Y^{k+1}{\left|\right|}_{\infty }\leq \left|\sum_{j=1}^{k}\left(b_{k-j}^{\left(\alpha \right)}-b_{k-j+1}^{\left(\alpha \right)}\right)Y_{i_{0}}^{j}+\mu \tau R_{i}^{k+1}\right|\\ \leq & |\sum_{j=1}^{k}\left(b_{k-j}^{\left(\alpha \right)}-b_{k-j+1}^{\left(\alpha \right)}\right){\left(b_{k}^{\left(\alpha \right)}\right)}^{-1}{\mu \tau |||R||}_{\infty }+{\mu \tau ||R||}_{\infty }\\ \leq & {\left(b_{k}^{\left(\alpha \right)}\right)}^{-1}{\mu \tau ||R||}_{\infty }\left(\sum_{j=1}^{k}\left(b_{k-j}^{\left(\alpha \right)}-b_{k-j+1}^{\left(\alpha \right)}\right)+b_{k}^{\left(\alpha \right)}\right)\\ \leq & {\left(b_{k}^{\left(\alpha \right)}\right)}^{-1}{\mu \tau ||R||}_{\infty }\left(b_{k-j}^{\left(\alpha \right)}-b_{k-j+1}^{\left(\alpha \right)}+b_{k}^{\left(\alpha \right)}\right)\\ \leq & {\left(b_{k}^{\left(\alpha \right)}\right)}^{-1}{\mu \tau ||R||}_{\infty }\\ \leq & C(\tau^{2-\alpha }+h^{2}). \end{array} \end{array}$$ (17)

So, fractional-order numerical model (10) is convergent.

## Genetic algorithm combined with an approximate grid method(GA-AGM)

In 1975, John Holland and others developed the genetic algorithm (GA). GA is an adaptive, global optimization search algorithm that simulates the process of biological inheritance and evolution in nature and has been applied to many fields of optimization and search. GA initializes a population first, then evaluates the fitness of individuals in the population, generates new individuals by selecting, crossing and mutating the population, and gradually optimizes individuals in the population to obtain the optimal solution. However, GA is prone to local optimization because the search space is reduced after crossover and mutation of the population, making it difficult to obtain the global optimal solution. Therefore, to avoid this defect, we first use an approximate grid method [25] to estimate the parameters in the model of this article. This can shorten the range of optimal parameter values. We then use GA to estimate the optimal parameters in the model.

Step 1: Let (*p*_1_, *p*_2_, ⋯, *p*_*m*_) ∈ *E*, where *E* is a bounded domain of the form

$$\begin{array}{c} E=\left[p_{1}^{\left(min\right)},p_{1}^{\left(max\right)}\right]\times \left[p_{2}^{\left(min\right)},p_{2}^{\left(max\right)}\right]\times \cdots \times \left[p_{3}^{\left(min\right)},p_{3}^{\left(max\right)}\right]. \end{array}$$ (18)

The intervals $\left[p_{j}^{\left(min\right)},p_{j}^{\left(max\right)}\right]\left(j=1,2,\cdots ,m\right)$ are first partitioned with step *h*_*j*_ and so

$$\begin{array}{c} p_{j}^{\left(min\right)}=p_{j,0}<p_{j,1}<\cdots <p_{j,M_{j}}=p_{j}^{\left(max\right)}, \end{array}$$ (19)

where $p_{j,k_{j}}=p_{j}^{\left(min\right)}+k_{j}\times h_{j}$ and $h_{j}=\frac{p_{j}^{\left(max\right)}-p_{j}^{\left(min\right)}}{M_{j}}$.

Let *P*^*min*^ = (*p*_1,0_, *p*_2,0_, ⋯, *p*_*m*,0_), then the grid *G*(*E*) is defined as

$$\begin{array}{c} G\left(E\right)=\left\{P\in E:p_{j,k_{j}}=p_{j}^{\left(min\right)}+k_{j}\times h_{j},j=1,2,\cdots ,m,k_{j}=0,1,\cdots ,M_{j}\right\}. \end{array}$$ (20)

With the above grid *G*(*E*), the approximate estimation of the unknown parameter vector $P^{*}=\left(p_{1}^{*},p_{2}^{*},\cdots ,p_{m}^{*}\right)\in G\left(E\right)$ is determined by the root-mean-square error function:

$$\begin{array}{c} g\left(P^{*}\right)=\min_{P\in G(E)}g\left(P\right)=\min_{P\in G(E)}\{\sqrt{\frac{\sum_{j=0}^{N}{\left(x\left(t_{j}\right)-x_{j}\right)}^{2}}{N+1}}\}, \end{array}$$ (21)

where *x*(*t*_*j*_) is the numerical solution of the fractional system (1) for the given parameters *P* = (*p*_1_, *p*_2_, ⋯, *p*_*m*_), and *x*_*j*_ is the real data.

For this minimization, the initial points in the grid *G*(*E*) must be predetermined. If step *h*_*j*_ is too small, the number of points in the grid *G*(*E*) will be very large, which will require more time to compute *g*(*P*). Hence, after the estimates of the unknown parameters of $P^{*}=\left(p_{1}^{*},p_{2}^{*},\cdots ,p_{m}^{*}\right)\in G\left(E\right)$ have been obtained, we then define a new domain and apply GA to estimate the parameters again.

Step 2: A new bounded domain is defined as follows:

$$\begin{array}{c} E^{*}=\left[p_{1}^{*}-L*h_{1},p_{1}^{*}+L*h_{1}\right]\times \left[p_{2}^{*}-L*h_{2},p_{2}^{*}+L*h_{2}\right]\times \cdots \times \left[p_{m}^{*}-L*h_{m},p_{m}^{*}+L*h_{m}\right], \end{array}$$ (22)

where *L* is a positive constant (we can choose *L* = 1, 2, 3). Apply GA to estimate the parameter vector $P^{**}=\left(p_{1}^{**},p_{2}^{**},\cdots ,p_{m}^{**}\right)\in G\left(E\right)$. The *i*-th generation population is represented as $P_{i}^{**}\in E^{*}$. Then we convert $P_{i}^{**}$ to binary for selection, crossover and mutation operations to obtain the next generation of the population $P_{i+1}^{**}$. Then we evaluate the fitness of the individual P** in the population and evaluate it according to the following equation:

$$\begin{array}{c} g\left(E^{*}\right)=\min_{P\in E^{*}}g\left(P\right)=\min_{P\in E^{*}}\{\frac{\sum_{j=0}^{N}{\left(x\left(t_{j}\right)-x_{j}\right)}^{2}}{N+1}\}. \end{array}$$ (23)

Step 3: Compute the error ||P* − P**|| and *g*(P**). If ||P* − P**|| < *ε* or *g*(P**) < *δ*, where *ε* and *δ* are small error parameters, P** is the approximate estimate of the parameter vector we want to find. Otherwise, let P* = P** and $h_{j}=h_{j}^{\prime }$, and go to Step 2. The scheme is run until ||P* − P**|| and *g*(P**) are small enough, i.e., ||P* − P**|| and *g*(P**) are less than the given constants. P** is the parameter estimate obtained by the genetic algorithm. This process is completed using MATLAB software.

Based on the numerical solutions obtained from the model and experimental data, we will use GA-AGM to obtain the optimal values of the unknown parameters (chloride diffusivity *D*, fractional order *α*, surface chloride ion concentration *c*_0_) in the model. Let (*D*, *α*, *c*_0_) ∈ *E*, where *E* is a bounded domain of the form

$$\begin{array}{c} E=[0,10]\times [0,1]\times [0,2]. \end{array}$$ (24)

And *m* = 3, *M*_1_ = 10, *M*_2_ = 10, *M*_3_ = 20, so *h*_1_ = 1, *h*_2_ = 0.1, *h*_3_ = 0.1.

## Study area and organized data sets

**Fig 1:**
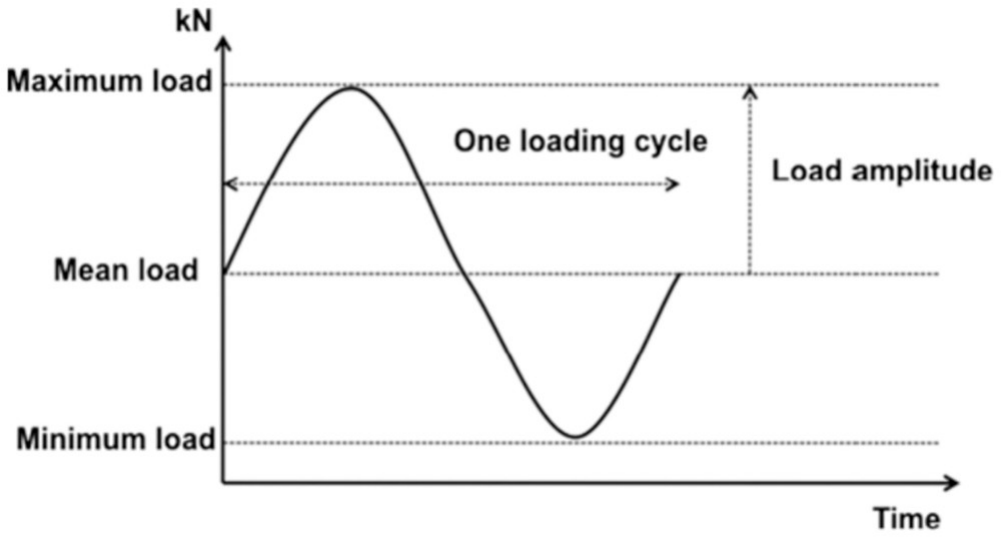


**Fig 2:**
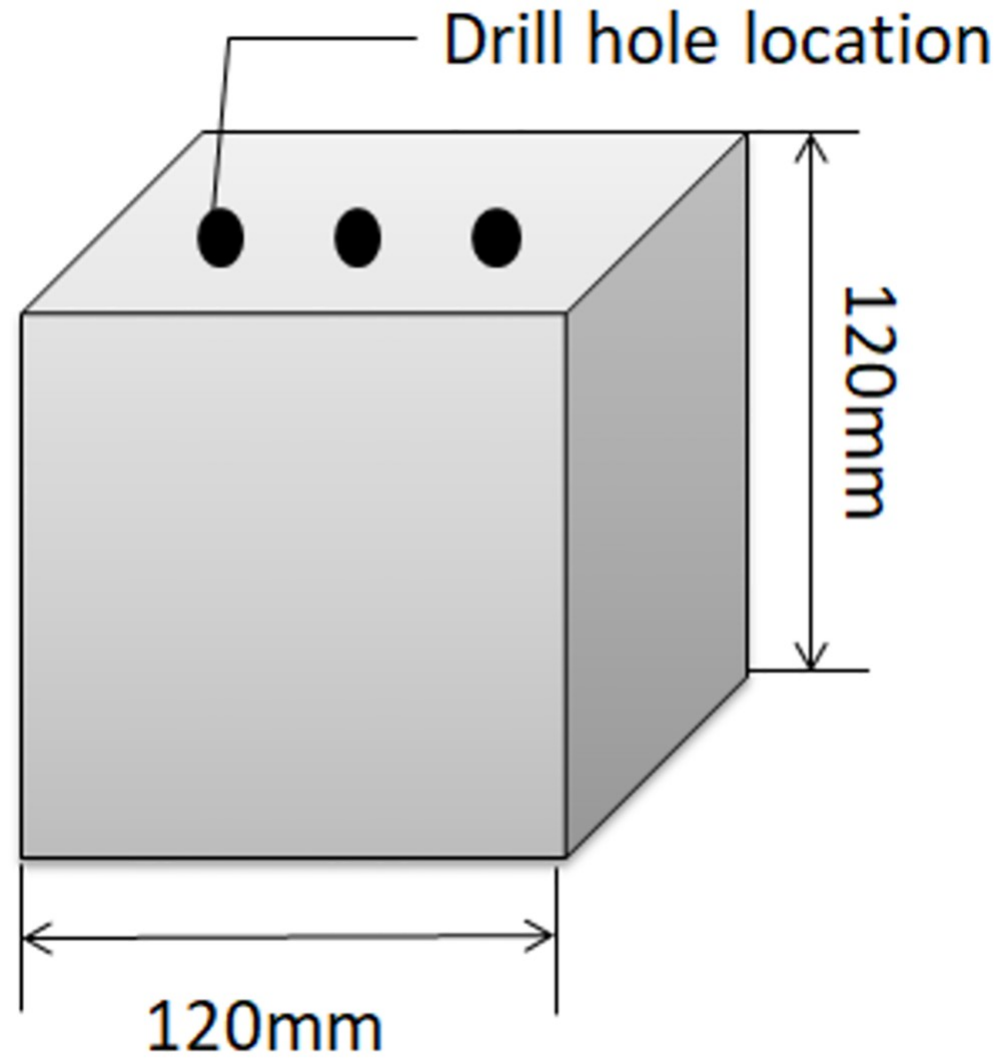


To verify the rationality of the fractional-order numerical model in this study, data from Chuanqing Fu et al. [26] were cited and compared with the numerical solution of the model. In their experiment, Chuanqing Fu et al. studied the effect of supplementary cementitious materials (i.e., blast furnace slag and class F fly ash) on chloride ion penetration in fatigue-damaged concrete. Three types of concrete were selected for the study: ordinary Portland cement concrete, ordinary Portland cement concrete with ground granulated blast furnace slag, and ordinary Portland cement concrete with class F fly ash, as shown in Table 1. The three types of concrete had the same water-binder ratio and paste-aggregate ratio. The mineral composition and fineness of ordinary Portland cement concrete are listed in Table 2. ASTMC989 grade 100 slag with a fineness of 450 *m*^2^/*kg* and ASTMC618 class F fly ash with a density of 2210 *kg*/*m*^3^ were selected. Three types of concrete with dimensions of 150 mm × 150 mm × 150 mm were used for tensile and compressive strength tests, and the compressive and tensile strengths are shown in Table 2. They conducted the fatigue-loading experiment in equal amplitude sinusoidal mode on three concrete specimens with dimensions of 120mm × 120mm × 1200mm. The constant amplitude sine loading mode is shown in Fig 1. After the fatigue loading test, each fatigue-loaded specimen was sawed into a cube with a side length of 120 mm. Then the cube specimens were coated with paraffin wax on all five sides of the specimens except for the side exposed to air in the experiment. The three concrete specimens were then placed in a wetting-drying cyclic environment and a fully submerged environment at 20°C. The wet and dry cyclic environment means that the specimens were immersed in 5% NaCl solution for six h per day and then dried in 40% RH for 18 h. The complete submersion environment means that the specimens were immersed in a solution filled with 5% NaCl solution for 24 h per day. The variation of chlorine content with depth was measured and recorded after 30, 45, and 60 days of testing in both environments for the three concrete specimens. The concrete powder was drilled at 2.5, 7.5, 12.5, 17.5, 22.5, 27.5, 35.0, and 45.0 mm on the concrete surface. Ten g of concrete powder was collected at each depth. And concrete specimen perforation location are shown in Fig 2. After the powder is dried and cooled, the chloride ion concentration is measured using a Thermo720 A, providing experimental data on the impact of blast furnace slag and Class F fly ash on the chloride ion penetration in fatigue-damaged concrete.

**Table 1:** Mineral composition (mass percentage) and fineness of ordinary silicate cement.

Chemical composition	C2S	C3S	C3A	C4AF	CaSO4⋅H2O	Purity (*m*^3^/*kg*)
Content	19.1	55.5	6.5	10.1	5	350

**Table 2:** Mixing ratio (*kg*/*m*^3^) and mechanical properties of concrete.

Mixture ID	Cement	Water	Fine Aggregate	Coarse Aggregate	28-day compressive strength(MPa)	28-day tensile strength(MPa)	Slag	Fly ash
PC	372	175	698	1116	41.6	2.65	0	0
SL	223	175	698	1116	36.7	2.31	149	0
FA	260	175	698	1116	34.2	2.26	0	112

## Discussion and results

### Model validity

**Fig 3:**
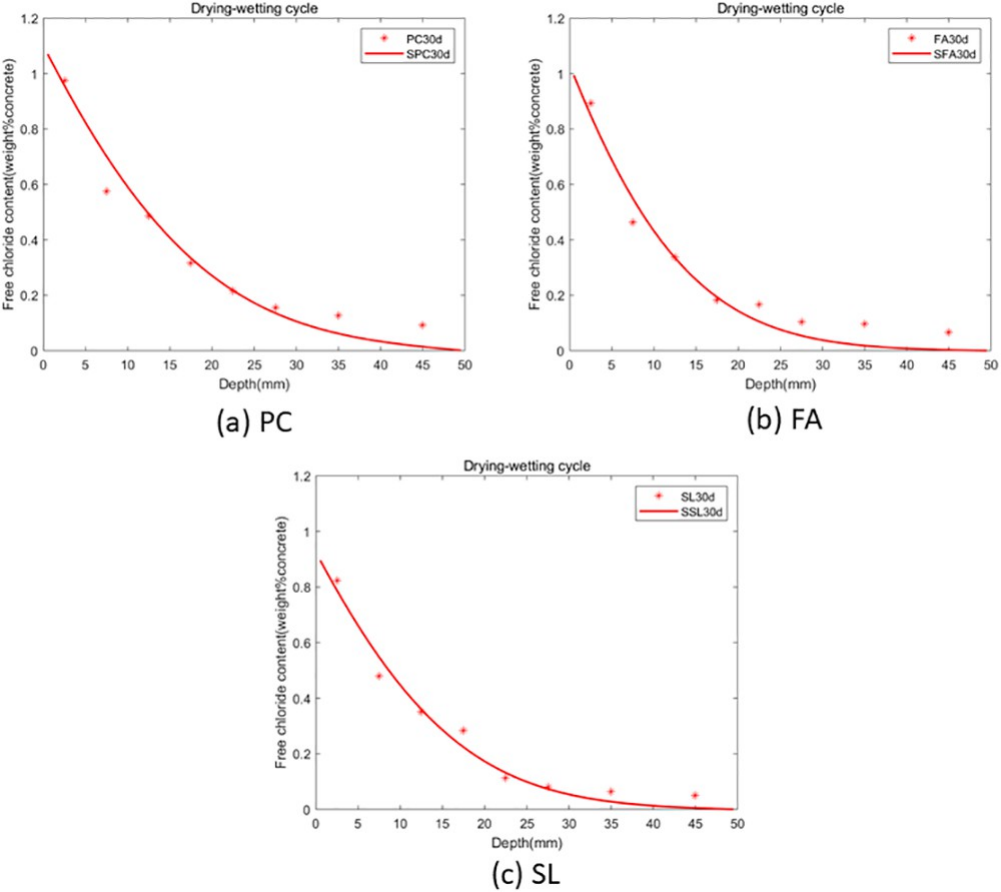


**Fig 4:**
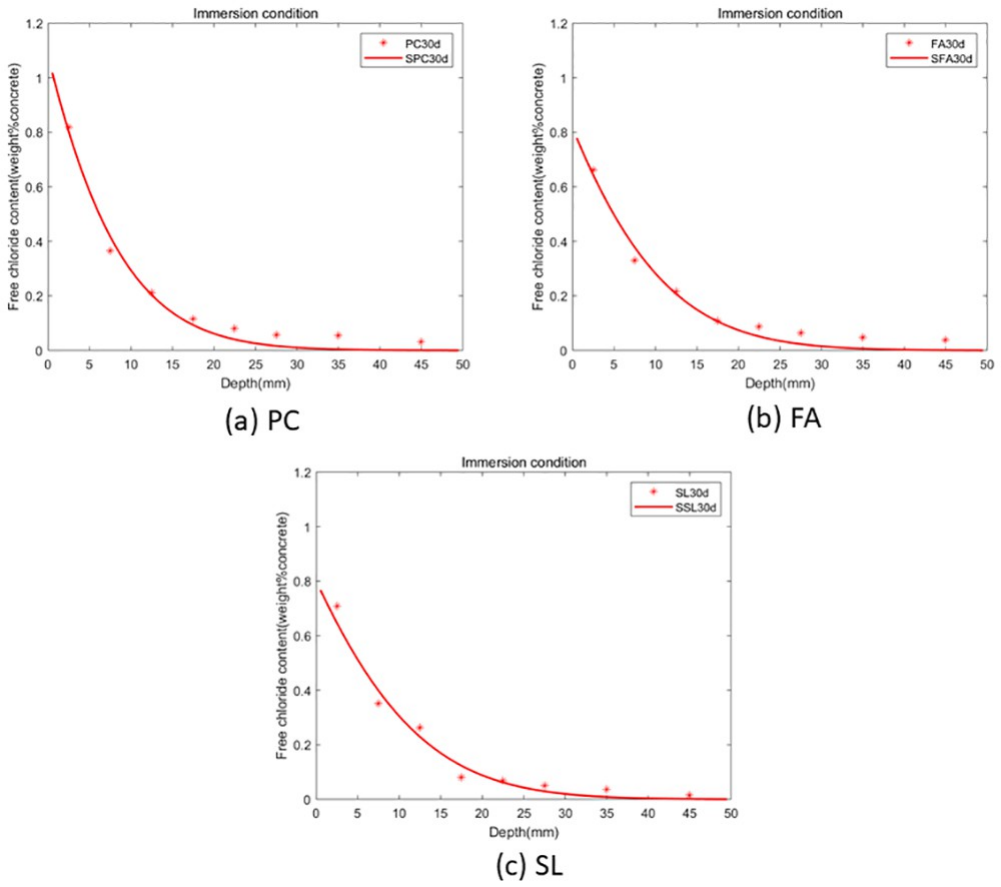


**Fig 5:**
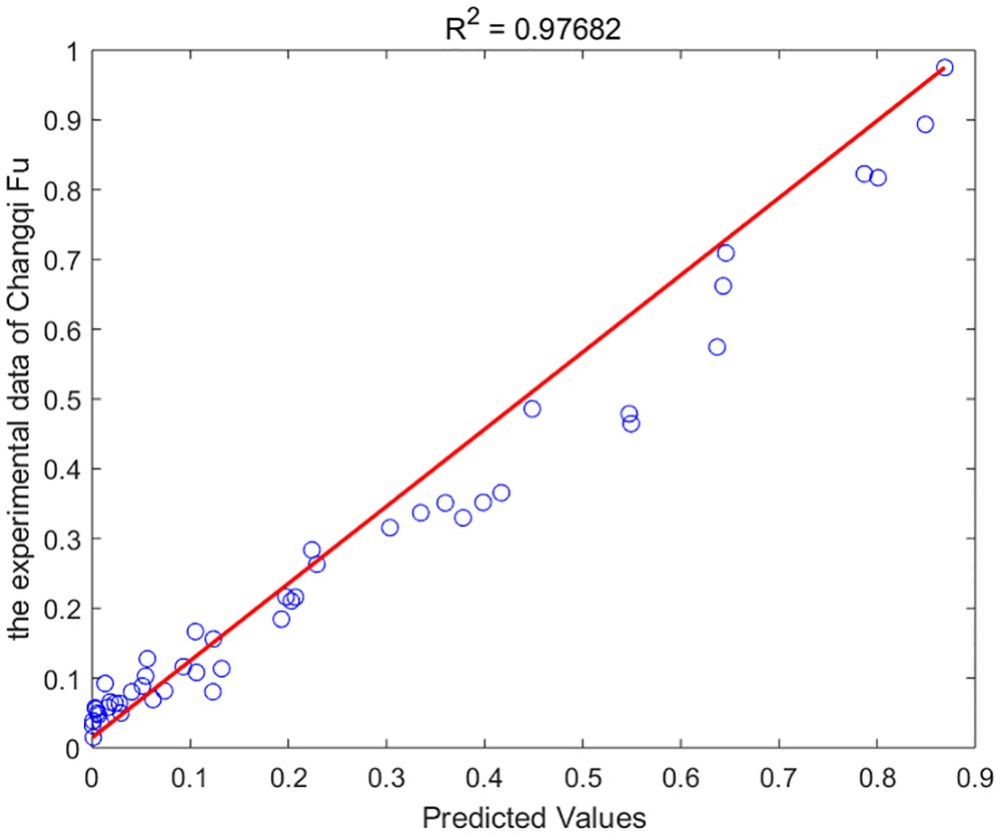


As per the theorem outlined in the preceding section, the fractional-order numerical scheme presented is unconditionally stable and convergent. Consequently, the model is applicable for any value of *α*. The GA-AGM was employed to determine the optimal values of the unknown parameters in the model, namely chloride diffusivity (*D*), fractional order (*α*) and surface chloride ion concentration *c*_0_. The optimal parameters are detailed in Tables 3 and 4. A comparison of the fractional-order numerical model’s fitting effect to the distribution of chloride ion content under drying-wetting cycle and immersion conditions with discrete test values is depicted in Figs 3 and 4. “PC” refers to the experimental data of chloride ion content in ordinary portland cement concrete. “FA” represents the experimental data of chloride ion content in concrete made with ordinary portland cement mixed with ground granulated blast furnace slag. “SSL” denotes the numerical solution of chloride ion content in concrete made with ordinary portland cement mixed with Class F fly ash. “SPC” stands for the experimental data of chloride ion content in ordinary portland cement concret. “SFA” refers to the numerical solution of chloride ion content in concrete made with concret mixed with ground granulated blast furnace slag. “SSL” again represents the numerical solution of chloride ion content in concrete made with concret mixed with Class F fly ash. The goodness-of-fit dicators are shown in Fig 5. As shown in in Fig 5, the fitting effect of the predicted chloride ion content in concrete corroded under dry-wet cycle and complete immersion conditions for 30 days with the experimental value is *R*^2^ = 0.976819. Therefore, the calculated values from the fractional-order numerical scheme align closely with experimental values, underscoring the model’s efficacy in simulating the permeation process of chloride ions in concrete with fly ash or slag.

**Table 3:** Estimates of unknown parameters under dry-wet cycle conditions.

unknown parameters	PC-30d	FA-30d	SL-30d	PC-45d	FA-45d	SL-45d	PC-60d	FA-60d	SL-60d
*α*	0.819	0.892	0.972	0.758	0.84	0.839	0.845	0.9	0.919
*D*	10	9	8	7	6.741	7.589	7	6	6.494
*c*_0_	1.1	1.032	0.924 1.185	0.97	1	1.305	1.2	1.063	

**Table 4:** Estimates of unknown parameters under immersion conditions.

unknown parameters	PC-30d	FA-30d	SL-30d	PC-45d	FA-45d	SL-45d	PC-60d	FA-60d	SL-60d
*α*	0.595	0.815	0.669	0.706	0.782	0.867	0.759	0.765	0.851
*D*	6.242	5	8.5	6	4	4	5.824	3.5	4.5
*c*_0_	1.08	0.816	0.8	1.037	0.9	0.989	1.129	0.968	0.967

### Model comparision

To verify that the Caputo time fractional-order numerical scheme simulates the chloride ion transport process better than the integer-order numerical scheme and the analytical solution of Fick’s second law. We first discretize the classical Fick’s second law diffusion equations $\frac{\partial u(x,t)}{\partial t}=\;D\frac{\partial^{2}u\left(x,t\right)}{\partial x^{2}}$. The initial and boundary values of the equation are (2) and (3). The discretization method is the same as the method used in Section 2. Then we can obtain the integer-order numerical scheme:

$$\begin{array}{c} \begin{array}{c} \left(1+\frac{2D\tau }{h^{2}}\right)u_{i}^{k+1}=u_{i}^{k}+\frac{D\tau }{h^{2}}\left(u_{i+1}^{k+1}+u_{i-1}^{k+1}\right),\\ i=1,2,\ldots ,m-1;k=1,2,\ldots ,n-1. \end{array} \end{array}$$ (25)

$$\begin{array}{c} \begin{array}{c} u_{i}^{0}=0,i=0,1,\ldots ,m,\\ u_{0}^{k}=\varphi_{0}\left(t_{k}\right),u_{m}^{k}=0,k=1,2,\ldots ,n. \end{array} \end{array}$$ (26)

The analytical solution of Fick’s second law [22]:

$$\begin{array}{c} \begin{array}{c} u\left(x,t\right)=u_{0}^{0}+\left(u_{0}^{k}-u_{0}^{0}\right)\left(1-erf\left(x/2\sqrt{Dt}\right)\right). \end{array} \end{array}$$ (27)

**Fig 6:**
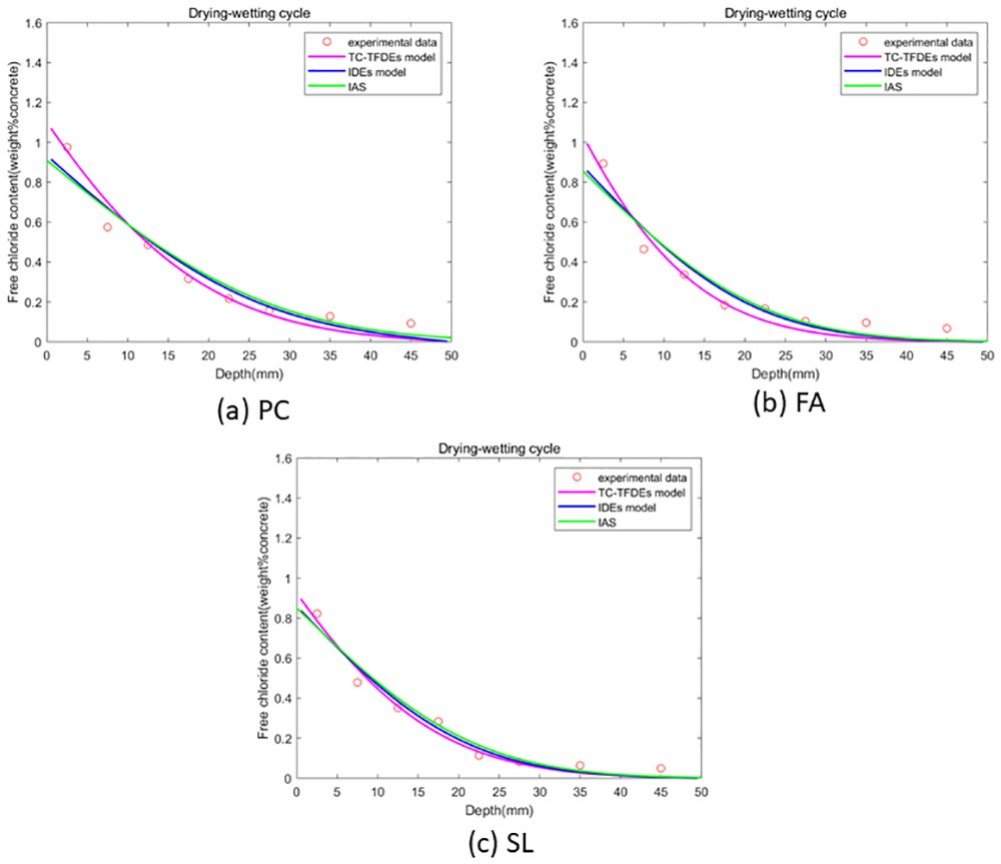


**Fig 7:**
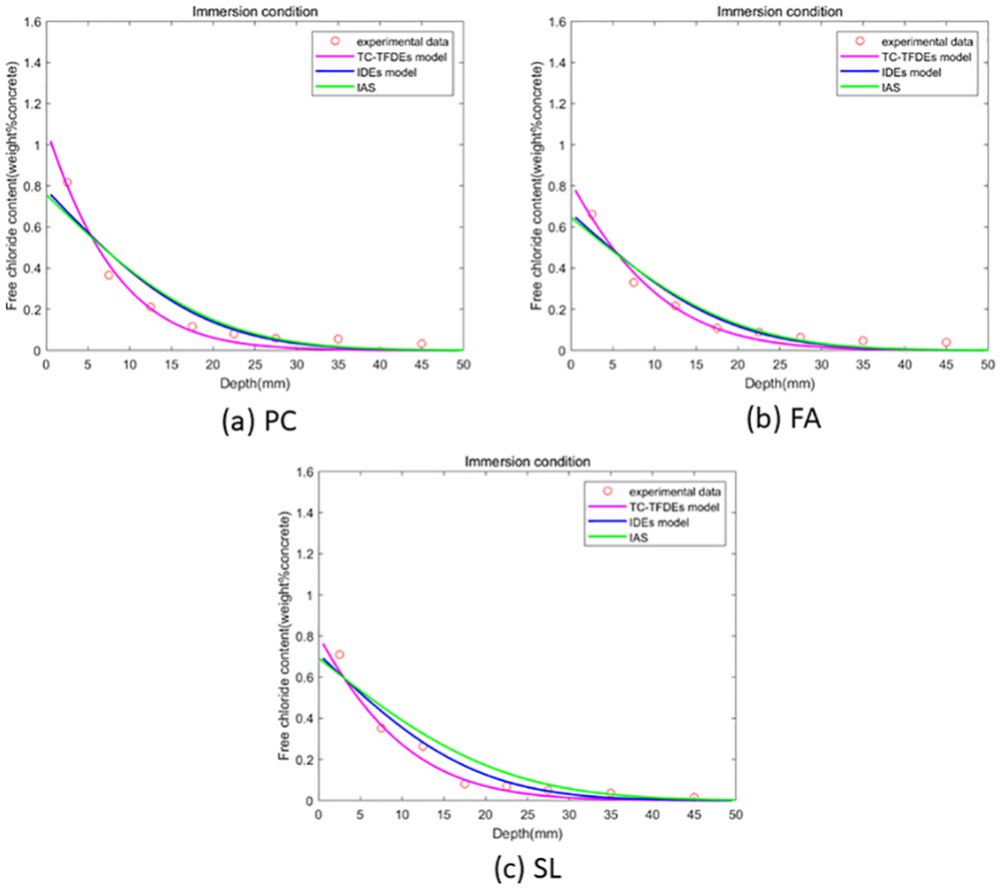


**Fig 8:**
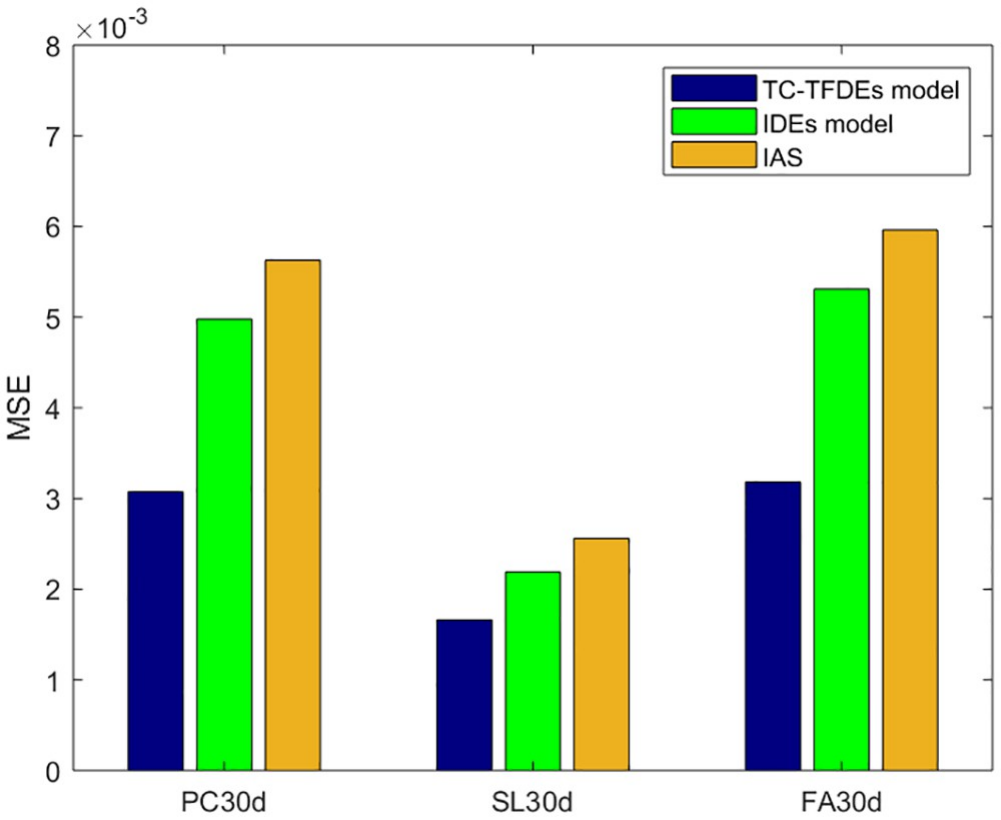


**Fig 9:**
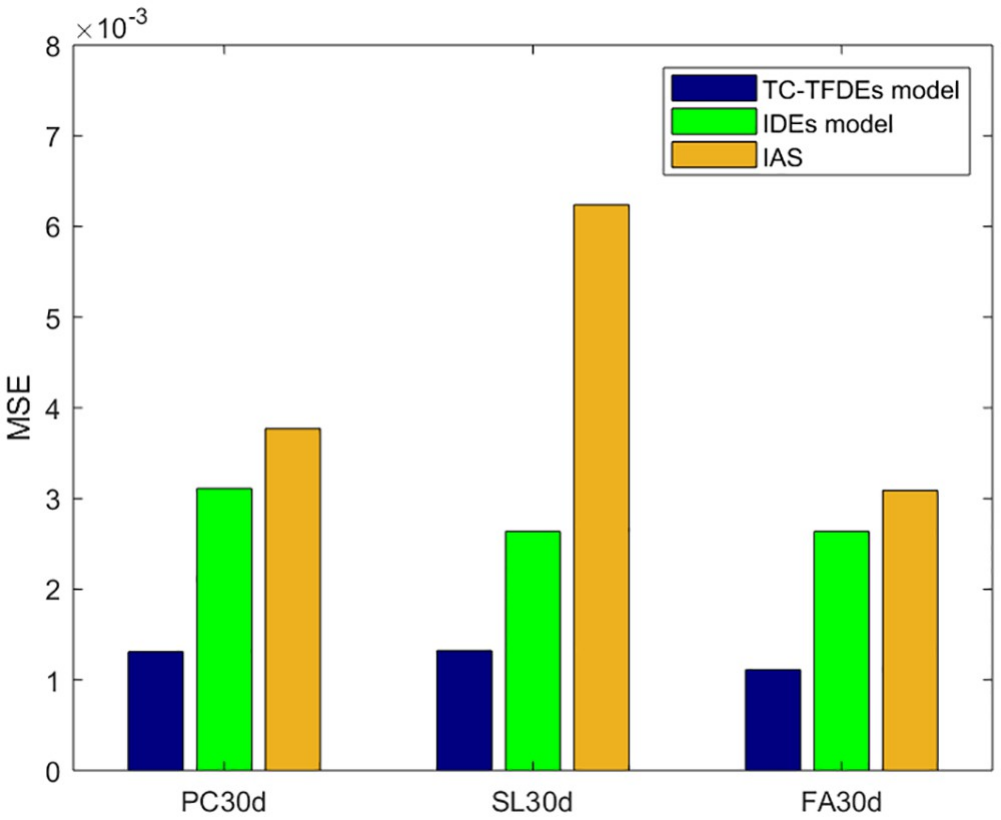


The optimal solution for the unknown parameters (chloride diffusivity *D*, fractional order *α*, surface chloride ion concentration *c*_0_) of the integer-order numerical format and analytical solution of the Fick’s second law diffusion equation is obtained using GA-AGM. The error range is kept consistent during the GA-AGM optimization process for the three models’ unknown parameters. The comparisons of the three model fitting effect under drying-wetting cycle and immersion conditions are shown in Figs 6 and 7. From Figs 6 and 7, we can see that all three models have good fitting effects, which verifies their effectiveness in simulating chloride ion transport in fly ash or slag concrete. To further verify that the fractional-order numerical scheme can better simulate chloride ion transport than the numerical solution and analytical solution of the classical Fick’s second law, the mean square error between the numerical solutions of the three models and experimental values is calculated, as shown in Figs 8 and 9. It can be seen from Figs 8 and 9 that the mean square error of the fractional-order numerical format proposed in this paper is smaller than that of the numerical solution and analytical solution of the classical Fick’s second law. Therefore, the numerical results obtained by the fractional-order numerical scheme can better simulate the distribution of chloride ions in concrete with fly ash and slag than the numerical solutions and analytical solutions of integer-order numerical scheme.

### Chloride ion transport

**Fig 10:**
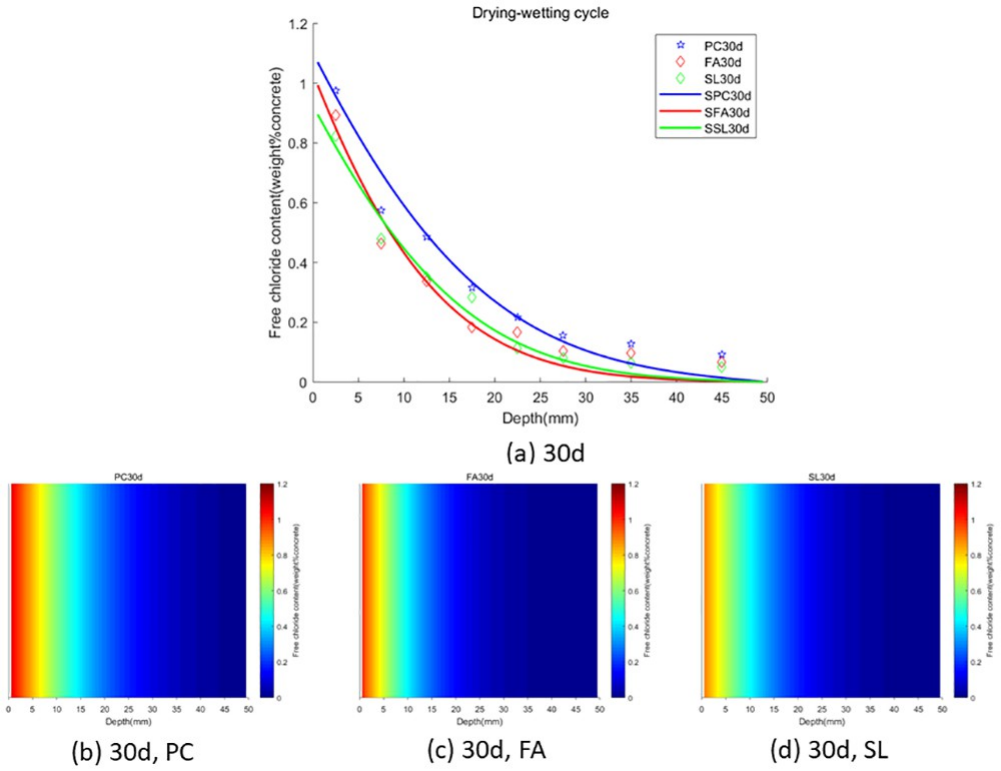


**Fig 11:**
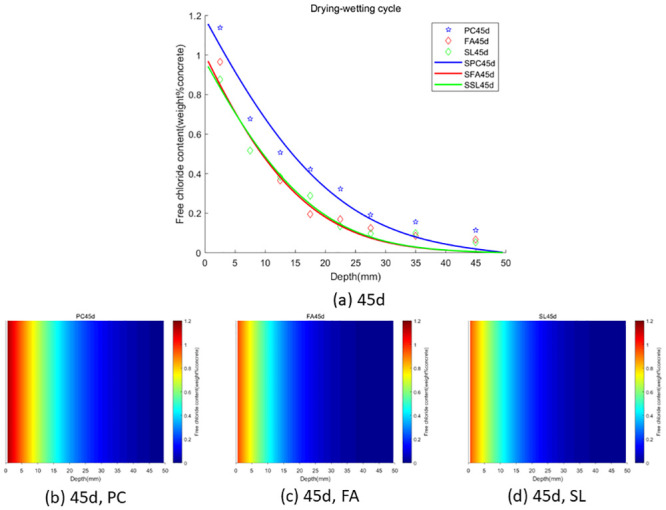


**Fig 12:**
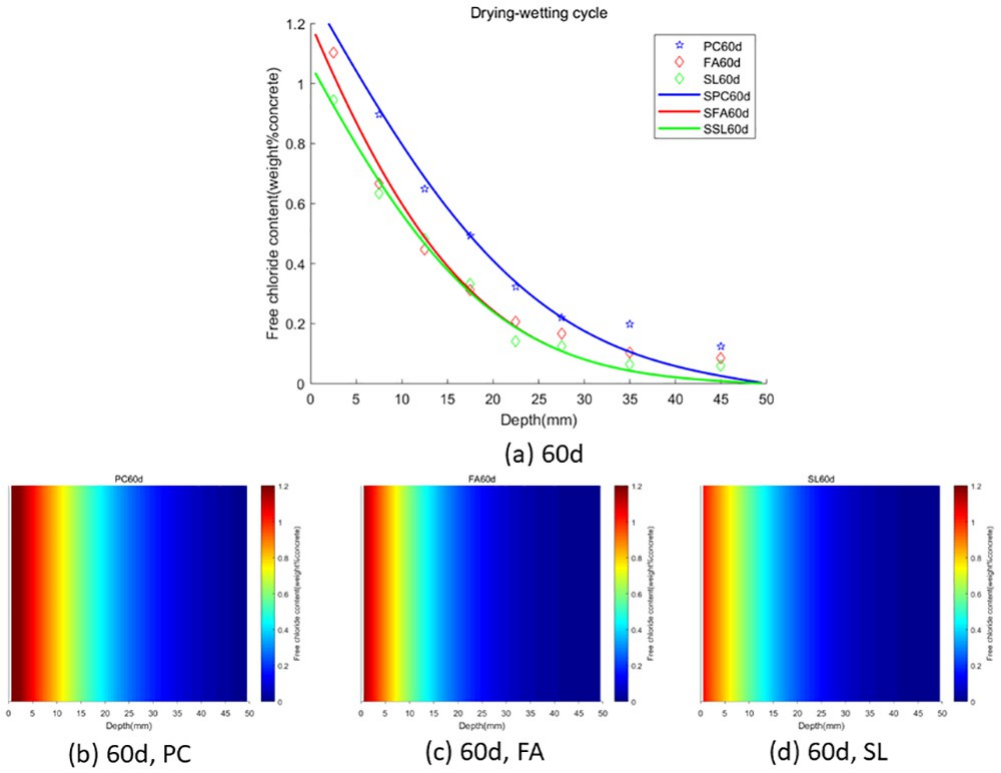


**Fig 13:**
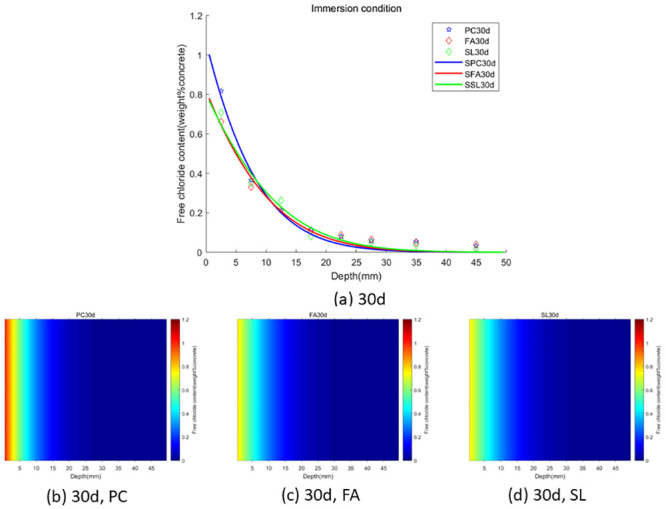


**Fig 14:**
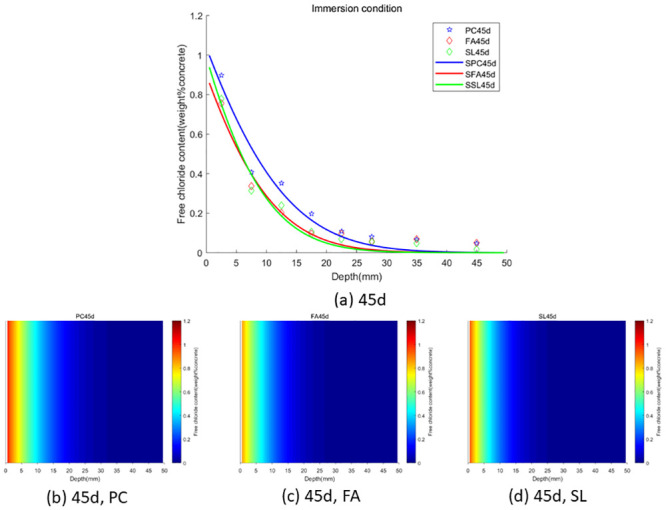


**Fig 15:**
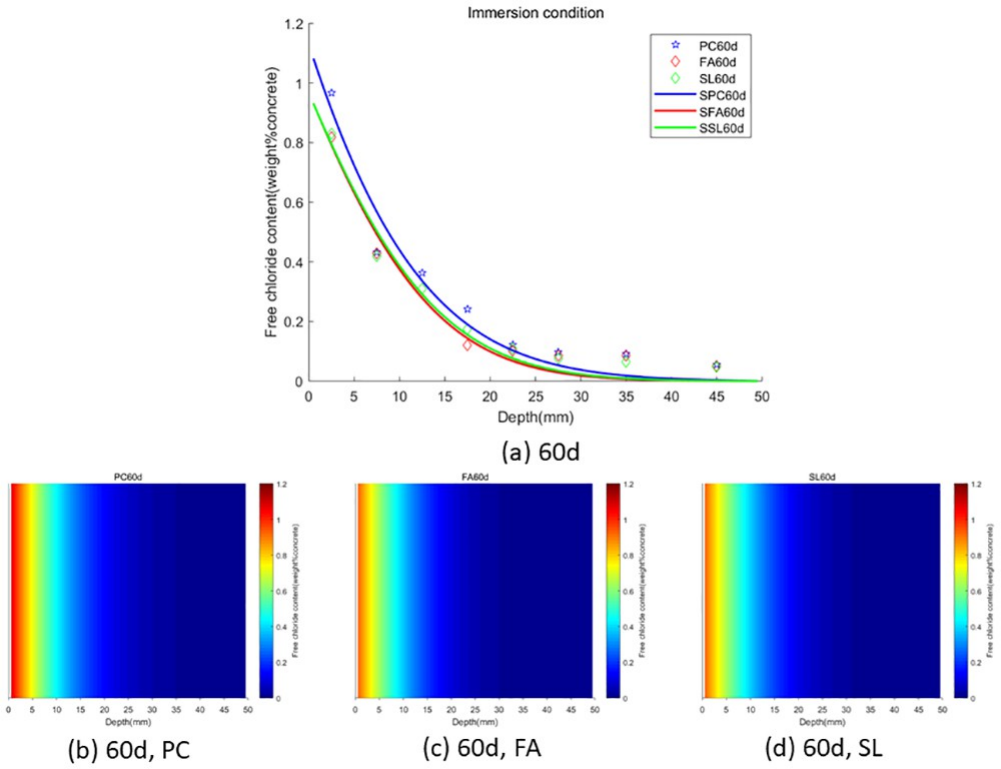


In the case of wet-dry cycles, chloride ions are mainly transported in concrete by diffusion and convection. In the case of complete immersion, chloride ions are mainly transported in concrete by permeation. To facilitate the observation of chloride ion transport in fly ash or slag concrete under two environments (wet-dry cycles and complete immersion), we used the Caputo fractional-order numerical model for numerical simulation and simulated the distribution map of chloride ions in concrete. We used GA-AGM to obtain optimal values of unknown parameters (chloride diffusivity *D*, fractional order *α*, surface chloride ion concentration *c*_0_) in the fractional-order numerical model, as shown in Tables 3 and 4. The content and distribution of chloride ions under dry-wet cycling and immersion conditions are shown in Figs 10 to 15. The red proportion of distribution maps b, c, and d from Figs 10 to 15 shows that the chloride ion content in fly ash or slag concrete is significantly lower than that in ordinary concrete under wet-dry cycles and complete immersion conditions. Adding fly ash or slag can alleviate the erosion of chloride ions on concrete. Comparing Figs 10 with 13, Figs 11 with 14, and Figs 12 with 15, the chloride ion content under wet-dry cycles is higher than that under complete immersion at the same erosion time. Under wet-dry cycle conditions, chloride ions mainly corrode concrete through diffusion and convection.

## Conclusions

1. Based on the classical Fick’s second law, Caputo time fractional derivative is introduced, and Caputo time fractional diffusion equation is established. The implicit numerical method is used to discretize the equation, and a fractional-order numerical format is established. Through theoretical research, it is proved that the fractional-order numerical format established in this paper has convergence and stability. GA-AGM estimates the unknown parameters of the fractional-order numerical format. The numerical results of the model fit well with Chuangqi Fu’s experimental data and the *R*^2^ value verifies the rationality of the model.
2. The Caputo time fractional numerical format is more in line with actual data results than the integer-order Fick’s second law numerical format and analytical solution fitting. The model established in this paper can better simulate chloride ion transport in fly ash or slag concrete and future service life.
3. Through analysis of the numerical simulation results, adding fly ash or slag can help to slow down the corrosion of chloride ions on concrete. Under dry-wet cycle conditions, chloride ion erosion on concrete is more severe than under immersion conditions.
